# Reimagining the United States organ procurement and transplant network

**DOI:** 10.3389/frtra.2023.1178505

**Published:** 2023-10-30

**Authors:** Eric Perakslis, Brian McCourt, Stuart Knechtle

**Affiliations:** ^1^ Duke Clinical Research Institute, Duke University School of Medicine, Durham, NC, United States; ^2^ Department of Surgery, Duke Transplant Center, Durham, NC, United States

**Keywords:** transplant, organ procurement transplant network, platform, business, modernization

## Abstract

The United States system of solid organ transplantation is overseen by the Organ Procurement Transplantation Network (OPTN). Recent announcements from the Health Resources and Services Administration (HRSA) indicate their clear intention to reform the system. We suggest that the original intention of the National Organ Transplant Act (NOTA) to require one entity to oversee transplantation is critical to integrate policy with the complex realities of organ procurement and transplantation practice. We suggest that a contemporary business platform model best captures the appropriate structure for coordinating organ transplantation, as the seamless exchange of organs between related groups is the essential function to facilitate. A business platform framework that includes public and private, academic and industry partners can best accomplish the important goal of equitable and efficient organ transplantation.

## Introduction

We have considered the widespread criticisms of the management of the U.S. organ transplant system by United Network for Organ Sharing (UNOS), and have suggested an approach to modernizing the information platform in a recent publication (1).

The weaknesses of the current contractor, UNOS, are significant, yet some approaches to change could make an already impossibly complex system far worse. Addressing the shortfalls of organ transplantation will require a modern platform business model, smart regulatory changes, and real-time data transparency. Briefly, a platform business is a business model that facilitates interactions between a large number of partners by helping participants learn and engage with each other faster than by using traditional business models. A governance model that sets standards and protocols can facilitate interactions at a much larger scale, allowing network effects that magnify efficiency.

The federal government will soon make the first significant regulatory and contracting changes to the OPTN since 1984 (2). Recent senate inquiries and a 2.5 year investigation have criticized OPTN performance, in particular the performance of the private, non-profit organization tasked with running the OPTN, UNOS. Federal inquiries have exposed deficiencies in operation of the U.S. transplantation system including discarded organs (1 in 5 kidneys), patient safety issues, variable performance from the 57 regional organ procurement originations (OPOs) and substantial disparities by race and geography, leaving transplantation out of reach of some of the most vulnerable patients. For example, the organ discard rate in the U.S., 17.9% for kidneys, compared unfavorably with France where the discard rate was 9.1% between 2004 and 2014 and the authors concluded that 62% of the kidneys discarded in the U.S. would have been transplanted in France (3). Briefly, the National Academy of Science, Engineering, and Medicine (NASEM) report specifies the need for modernizing the information technology infrastructure and data collection for organ procurement, allocation, and distribution; improving equity, process of policy-making, and embedding quality improvement.

The recent Modernization Initiative announced by the Health Resources Services Administration (HRSA) outlines dissolution of the single contractor approach as specified by NOTA). We applaud HRSA taking action to strengthen accountability and transparency of the OPTN and actions to modernize the information technology and data collection and management process of the OPTN. Our hope and intention is to promote the significant change necessary to be truly responsive to the recommendations outlined in the NASEM report. The forthcoming contracts, however structured, are an opportunity to reinvent how transplant is administered in the United States for decades to come, not simply resolve today's deficiencies. We believe a strong, centralized, business platform model—that leverages successful examples outside of healthcare—with highly federated yet explicitly defined system of stakeholder interactions will be superior to a simply upgraded information system. Here's why.

## Policy options and implications

### The original vision was well-conceived and remains relevant

While execution and evolution over time may have floundered, the original vision of the OPTN was smart and well-conceived. Following NOTA in 1984, Health and Human Services (HHS) codified the requirements and operating principles of the OPTN via Final Rule March 16, 2000. Explicitly, the Purpose of the OPTN within the Final Rule states, “*to operate and monitor an equitable system for allocating organs donated for transplantation; maintain a waiting list of potential recipients…; match potential recipients with organ donors according to established medical criteria…, for listing and de-listing transplant patients; facilitate the efficient, effective placement of organs; and increase organ donation*”.

Flashing ahead 28 years and the current state of OPTN operations and technology are shown in the figures below taken from an independent research report supported by Arnold Ventures et al.

Clear in these diagrams are the complexity of parties involved, numerous iterative loops and the unoptimized mix of manual, technology-enabled and automated tasks. While complex, the actual volume, 41,354 organs transplanted in 2021, is modest compared to modern platform businesses as Amazon ships 66,000 orders per hour. In other words, a modern platform business such as Amazon achieves efficiencies at a scale that would be adaptable to the needs of organ transplantation by implementing business processes that are relevant and useful to the task of matching donors and recipients who are, from a business model perspective, analogous to sellers and buyers.

### Data, technology, process and logistics are inseparable in organ transplantation

One approach to the complexity of the OPTN would be to disaggregate the network into multiple peer contracts and contractors, each providing specific expertise and services. While leaning into simplicity is logical and attractive, it will be challenging to maintain integration and oversight after separation of the functions. When services consist of multiple simultaneous manual and automated processes, technology, operations, and logistics become inseparable.

**Figure 1:**
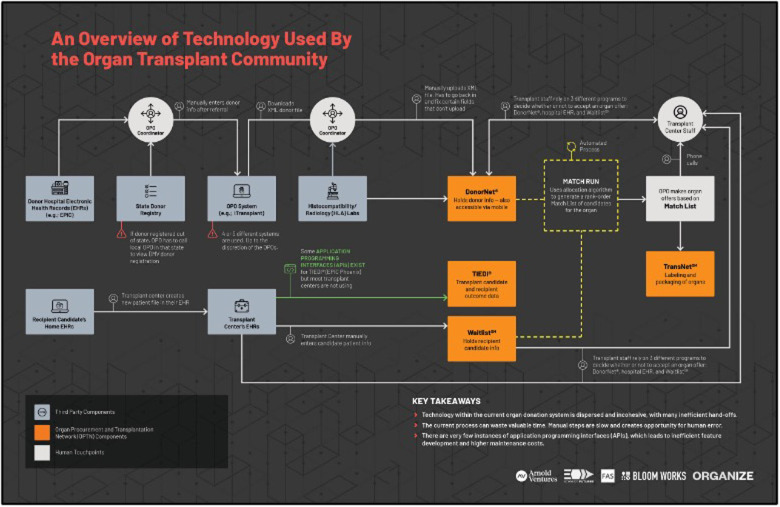
How an organ is managed (or not) in the current organ donation system.

**Figure 2:**
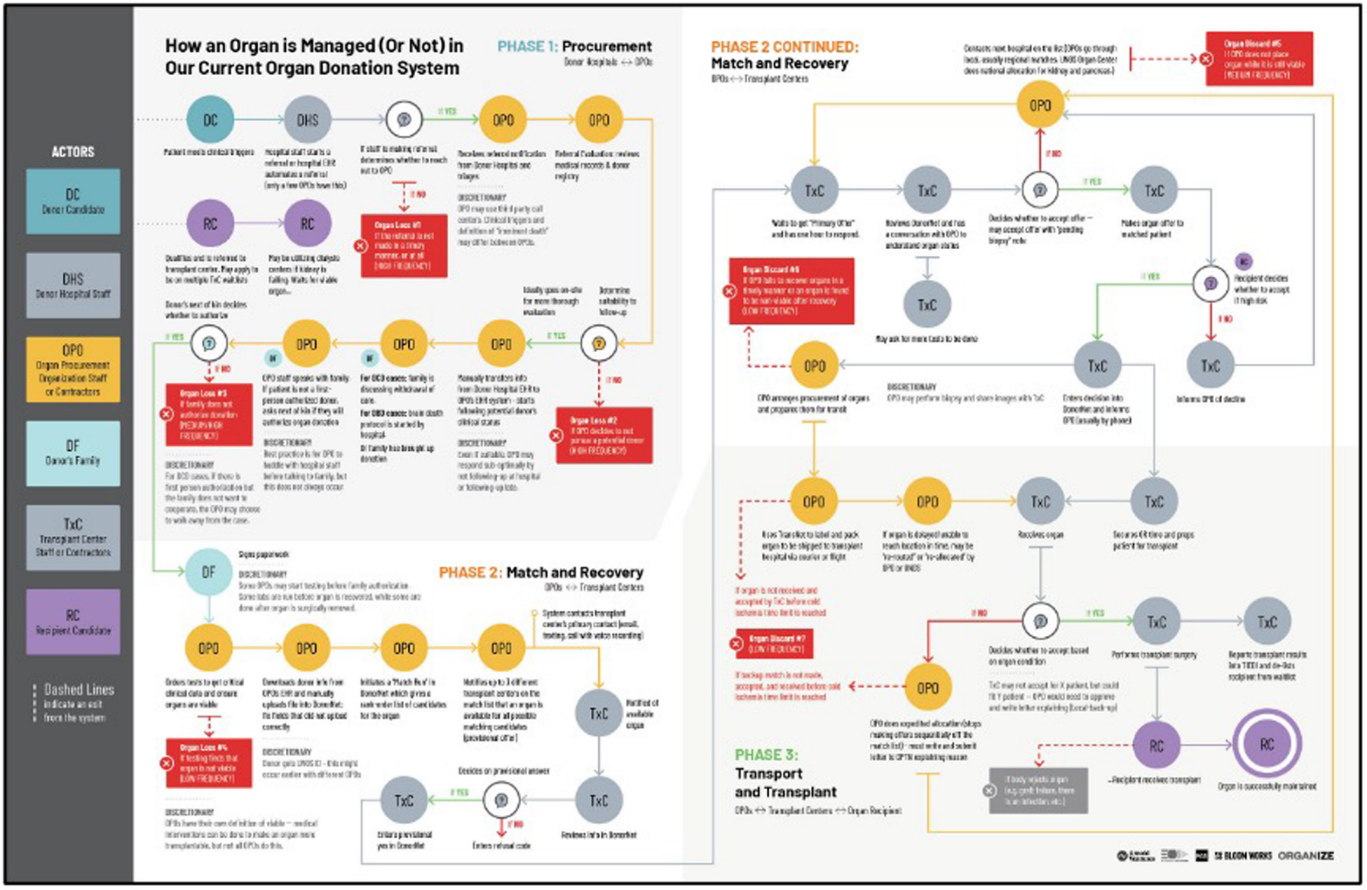
An overview of technology used by the organ transplant community.

Following the organ allocation process in Figure 1, the opportunities for digitization become clear but only if the technology and process are integrated, simultaneous, and optimized for low data latency. Today the process involves UNOS, 57 OPOs, 141 histocompatibility laboratories and more than 250 transplant centers. The technology and data being used across the OPOs has never been standardized. Everything from databases to shipping and logistics varies widely, and the fragmentation of processes and systems clearly contributes to the dysfunction of the overall system (Figure 2). Furthermore, policymaking happens at a slower pace than necessary for safety, often due to a lack of credible national-level data and due to excessively prolonged deliberation and review without preliminary testing of novel solutions.

### Data governance, availability and reduced data latency as core goals

While the investigation of OPTN point towards improved information technology (IT) systems, the core need is high quality, low latency data availability across the network. Table 1 presents the NASEM recommendations, the underlying data required to accomplish those recommendations and exemplars of successful examples.

**Table 1:** OPTN data and business model opportunities.

NASEM report recommendation	Essential data	Exemplars
Develop national performance goals for the U.S. organ transplantation system	Real-time & detailed data from OPOs and Transplant Centers	OMB Performance Measure Examples
Improve the OPTN policy-making process	Ability to experiment on “active” data and systems	Route Optimization for Shipping
Achieve equity in the U.S. transplantation system within 5 years	Social Determinants of Health (SDOH) and Health Status	STS National Database
Accelerate finalizing continuous distribution allocation frameworks for all organs	Real-time & detailed data from OPOs and Transplant Centers	Federal Aviation Administration. Air Traffic Control Towers
Eliminate predialysis waiting time points from the kidney allocation system	Detailed data on current wait times nationally	MyChart
Study opportunities to improve equity and use of organs in allocation systems	Longitudinal data on organ failure patients that are never listed	Kidney Failure Registries
Increase equity in organ allocation algorithms	Accurate and up-to-date centralized data for federated learning	Algorithmic Businesses
Modernize the IT infrastructure & data collection for deceased donor organ procurement, allocation, & distribution	Omni-channel data architecture and stakeholder experience	https://www.target.com/
Make it easier for transplant centers to say “yes” to organ offers	Timely and detailed acceptance/decline data from transplant centers	Shopify
Increase transparency & accountability for organ offer declines, & prioritize patient engagement in decisions regarding organ offers	Replace current ordinal process with transparency & opportunity via an open “bid” process for organs	EBay
Require the establishment & use of a donor care unit for each organ procurement organization	Geospatial, SDOH and medical status data on deceased donor occurrence regionally	CMS Oncology Care Model
Create a dashboard of standardized metrics to track performance & evaluate results in the U.S. organ transplantation system	Real-time, detailed performance data from OPOs & Transplant Centers	Amazon Seller Performance Team
Embed continuous quality improvement efforts across the fabric of the U.S. organ transplantation system	Six-month snapshots of actual performance data nationally	FedEx Distribution of Time Sensitive Materials
Align reimbursement and programs with desired behaviors and outcomes	Short & Long-term health outcomes data	National Cancer Institute SEER Database

It is essential to note that data availability and latency are typically social governance issues and not technological in nature. If data governance does not evolve to highly transparent levels, the opportunities in Table 1 will not be realized. In the case of the OPTN, improving data governance will require a re-balancing of accountabilities across the OPTN.

### The OPTN was envisioned as and remains a technology-based business

UNOS is actually named after the original computer program, the United Network for Organ Sharing, created in 1982 as a first automated system to assist with matching potential organ recipients with candidate organs that could not be used locally. Today, more than 60% of UNOS senior leadership team have information technology skillsets, titles and/or responsibility. In many ways, UNOS was envisioned and developed as one of the earliest incarnations of a platform business.

A platform is a business model that creates value by facilitating exchanges between two or more interdependent groups, usually consumers and producers. To make these exchanges happen, platforms harness and create large, scalable networks of users and resources that can be accessed on demand. Platforms create communities and markets with network effects that allow users and stakeholders to interact and transact.

## Actionable recommendations

### Understanding platform businesses

A key element of multi-sided platforms is that, instead of selling products, they facilitate exchanges between two or more related user groups. An example is how Uber does not provide taxi services, but rather they facilitate the exchange between a rider and a driver. Organs do not pass through UNOS today, but UNOS facilitates the exchange of organs between the Organ Procurement Organizations (OPOs) and transplant centers. Key to understanding platform businesses is that the technology is not the business but is the basis of the business and is inseparable. The business of Uber is connecting the rider and driver and the app is the toolsets that facilitates the relationship.

Similarly, FedEx has a national distribution system that efficiently delivers time-sensitive materials on next-day schedule as needed, utilizing their own coordinated transportation and tracking methods that we expect of current logistics technology. Organ transplants in contrast rely on *ad hoc* and locally implemented transportation systems with or without tracking tools.

The OPTN can clearly be envisioned as a modern, digital platform business and is best recreated by the technology platform remaining accountable and linked to the essential functions of the OPTN. Platform businesses have technology partners and service providers, but the basis of their operation is the development and execution of their core platform. This was evident early in the development of rideshare apps like Uber and Lyft. Traditional taxi companies sought to compete by developing their own mobile apps, which did make calling a taxi easier but ultimately failed as they could not replicate the efficiencies of a true platform business approach.

## Conclusions

### The missing elements: competition & performance

Parsing out the OPTN to multiple contractors can create the disruption required for transformative change, but it also risks compounding the current dysfunctions by fragmenting oversight and coordination. Competition is critical for the success of platform businesses. How would Uber look today without competition from Lyft? Striking a balance between strategy, process, policy, logistics and overall performance are equally unlikely without a competitive landscape. We need new ideas and new actors. We also need the information platform to support each of the essential functions of the OPTN, with the organ donor and transplant recipient central to the information platform and the functions revolving around their data.

The OPTN can be improved by strong, accountable, and centralized leadership and a platform business model is one compelling option for HRSA to consider as it modernizes the U.S. transplant system. In any case, patients are waiting.
